# Supplementary material for: Clarifying the Dominant Role of Crystallinity and Molecular Orientation in Differently Processed Thin Films of Regioregular Poly(3-hexylthiophene)
Source: Micromachines (Basel). 2024 May 22;15(6):677. doi: 10.3390/mi15060677 (PMC11205662; doi:10.3390/mi15060677)
Supplement: Supplementary file 1 [file micromachines-15-00677-s001.zip › micromachines-3011314-supplementary.pdf]

Supplementary Information

Clarifying the dominant role of crystallinity and molecular orientation in differently processed thin films regioregular poly(3-hexylthiophene)

Kumar Vivek Gaurav, Harshita Rai, Kshitij RB Singh, Shubham Sharma, Yoshito Ando, and Shyam S. Pandey*

Graduate School of Life Science and Systems Engineering, Kyushu Institute of Technology, Kitakyushu, Fukuoka, 808-0196 Japan

*Corresponding author (shyam@life.kyutech.ac.jp)

1. Optimization and Optical characterizations of spin-coated films

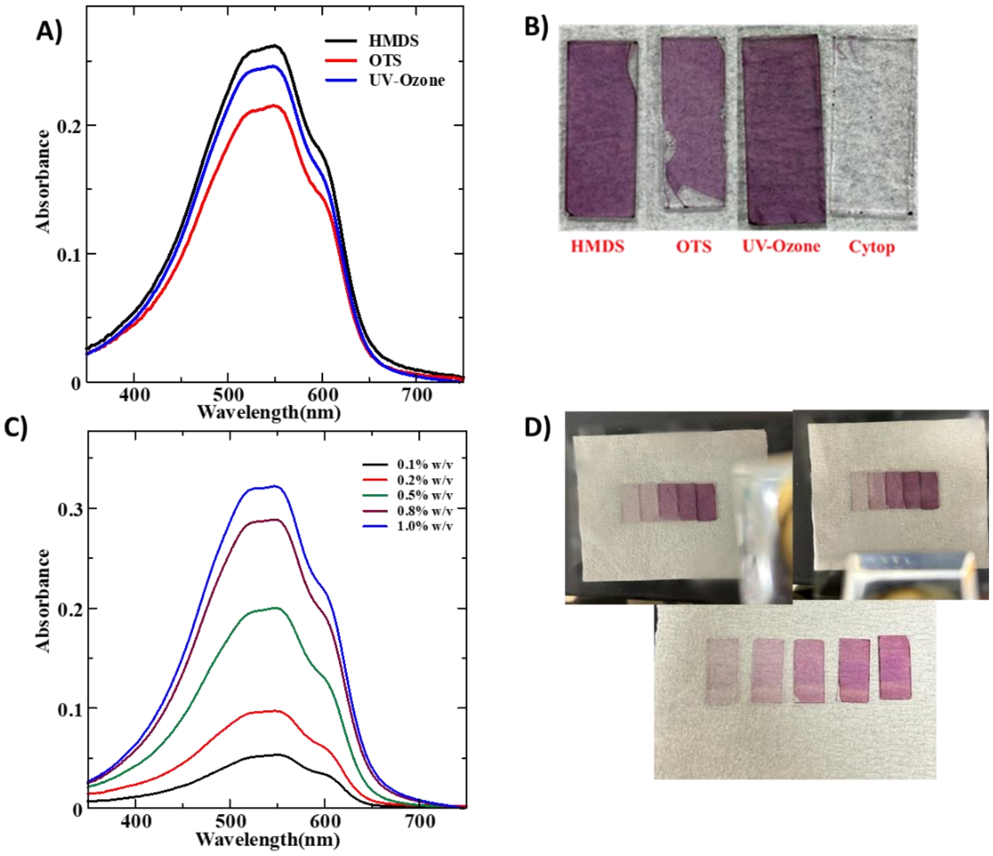

Figure S1. UV-Visible absorption spectra for spin-coated RR-P3HT thin films fabricated on differently surface-treated glass substrates A) and the photographs of corresponding thin films B). UV-Vis absorption spectra by varying the concentrations of RR-P3HT in Chloroform at a fixed spinning speed of 3500rpm on UV-Ozone treated glass substrate C) and the corresponding photographs spin-coated films with different concentrations under polarizer D) Polarized image of spin-coated films.

S2. Optimization and Optical characterizations of dip-coated films

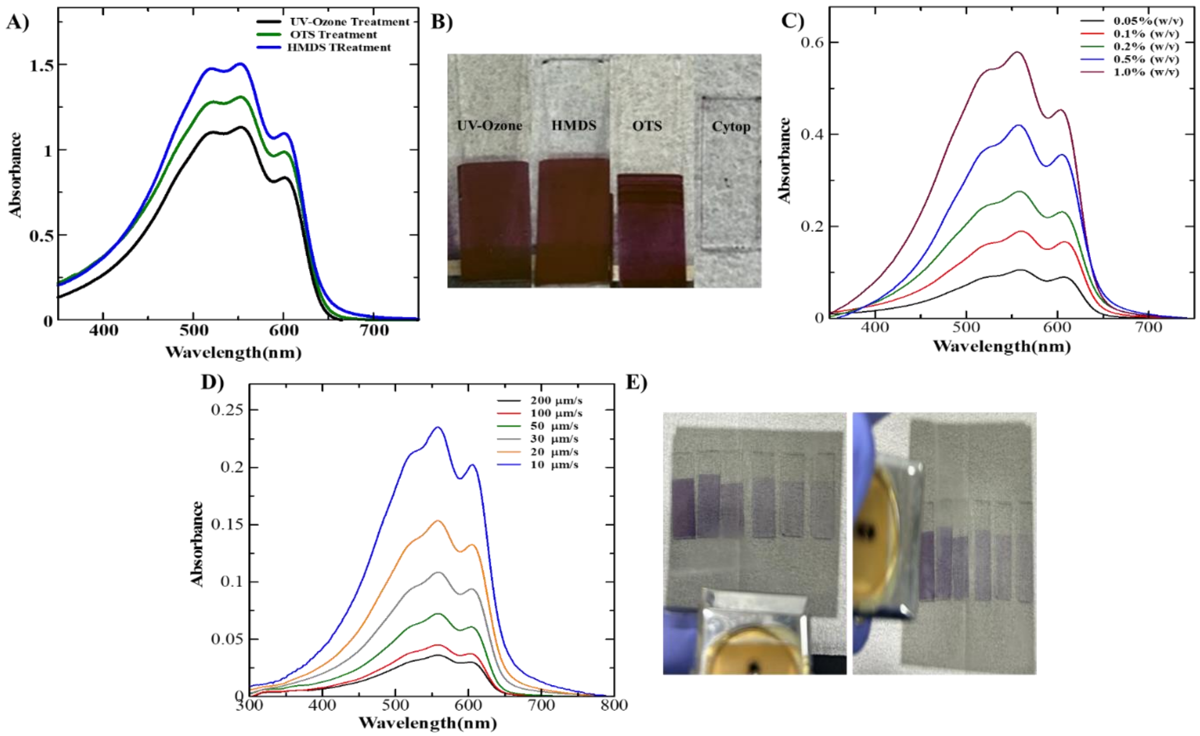

Figure S2. A) Electronic absorption spectra for dip-coated RR-P3HT thin films on glass substrate using different surface modifications at fixed lifting speed and concentration of 20 μm/s and 0.1 % (w/v), respectively and B) photographs of corresponding thin films. C) Absorption spectra on UV-Ozone treated glass substrate with varying concentrations of RR-P3HT in Chloroform at a fixed lifting speed of 20 μm/s. D) Absorption spectra on UV-Ozone treated glass substrate at various lifting speeds while keeping the polymer concentration of 0.1 % fixed and E) corresponding photographs of dip-coated thin films under polarizer.

S3. Optimization and Optical characterizations of FTM-processed thin films

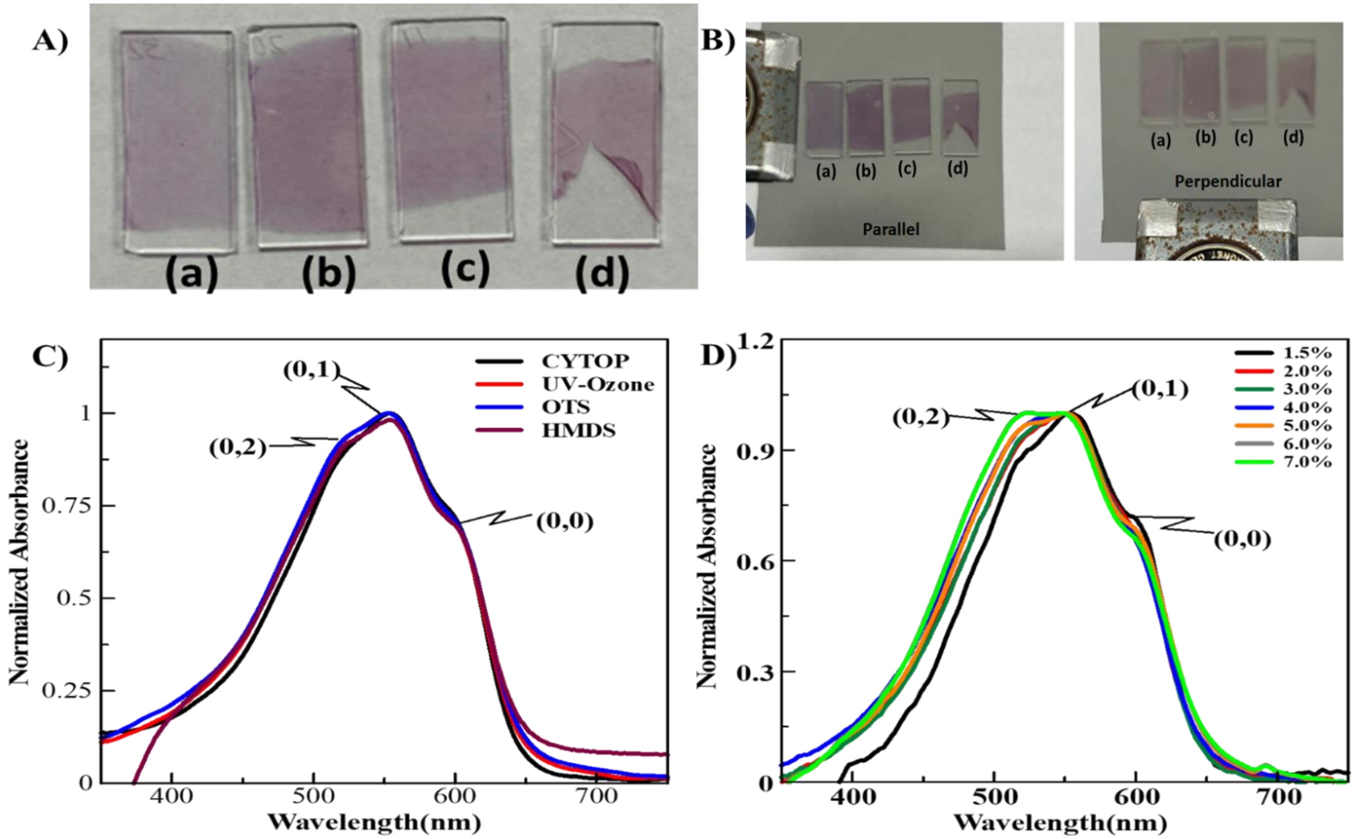

Figure S3. Photographs of the FTM-processed thin films prepared on differently surface-modified glass substrates without polarizer A) and with polarizer B), where a, b, c, and d represent glass substrates modified with CYTOP, OTS, HMDS and UV-Ozone, respectively. Normalized absorption spectra on differently surface-treated glass substrates C) and UV-Ozone treated glass substrates utilizing varying RR-P3HT concentrations.

Keywords: Regioregular poly(3-hexylthiophene); organic field-effect transistors; thin films; crystallinity; orientation; unidirectional floating film transfer
